# Engineering Microfluidic Organoid-on-a-Chip Platforms

**DOI:** 10.3390/mi10030165

**Published:** 2019-02-27

**Authors:** Fang Yu, Walter Hunziker, Deepak Choudhury

**Affiliations:** 1 Bio-Manufacturing Programme, Singapore Institute of Manufacturing Technology (SIMTech), Agency for Science, Technology and Research (A*STAR), 2 Fusionopolis Way, #08-04, Innovis, Singapore 138634, Singapore; fang_yu@simtech.a-star.edu.sg; 2 Institute of Molecular and Cell Biology (IMCB), Agency for Science, Technology and Research (A*STAR), 61 Biopolis Drive, Proteos, Singapore 138673, Singapore; hunziker@imcb.a-star.edu.sg; 3 Department of Physiology, 2 Medical Drive, MD9, National University of Singapore, Singapore 117593, Singapore

**Keywords:** organoids, microfluidics, organ-on-a-chip, microbioreactor, drug screening, cell culture

## Abstract

*In vitro* cell culture models are emerging as promising tools to understand human development, disease progression, and provide reliable, rapid and cost-effective results for drug discovery and screening. In recent years, an increasing number of *in vitro* models with complex organization and controlled microenvironment have been developed to mimic the *in vivo* organ structure and function. The invention of organoids, self-organized organ-like cell aggregates that originate from multipotent stem cells, has allowed a whole new level of biomimicry to be achieved. Microfluidic organoid-on-a-chip platforms can facilitate better nutrient and gas exchange and recapitulate 3D tissue architecture and physiology. They have the potential to transform the landscape of drug development and testing. In this review, we discuss the challenges in the current organoid models and describe the recent progress in the field of organoid-on-a-chip.

## 1. Introduction

Organoids are a new type of 3D culture models that have emerged in recent years. They are essentially miniaturized organs generated from stem cells *in vitro*. Under appropriate growth factor treatments, these cells differentiate and self-organize into organ-specific cell types and tissue organization. This allows recapitulation of the structure and function of the organs [1] and is useful for the study of human developmental biology and pathology. Organoid models have the potential to complement or even substitute for animal models. Compared with animal models, the organoid model can be scaled-up for high throughput testing at a lower cost with fewer ethical concerns. In addition, it can be used to study human diseases that are difficult to be modeled accurately in animals [2].

Organoids could be produced from various sources such as primary cells [3], pluripotent stem cells [4,5], embryonic [6] or adult stem cells [7], and patient-derived induced pluripotent stem cells [4,8]. Currently, there are three general approaches to generate organoid *in vitro*: (1) organoid formation on extracellular matrix (for example Matrigel) scaffolds [3]. As an example for this approach, a single Lgr5^+^ stem cell present at the tip of the intestinal crypt could self-organize into a crypt-villi structure and form intestinal an organoid without the support of mesenchymal tissue. (2) Organoid formation from embryoid body (EB) by agitation using spinning bioreactors [4]. This approach is often used to produce cerebral and retinal organoids. As demonstrated by Lancaster et al., skin fibroblast cells from a patient with microcephaly are reprogrammed using lentiviral delivery of the four major reprogramming factors, Oct4, Sox2, Klf4, and c-Myc. These induced pluripotent stem cells (iPSCs) form EBs, as an intermediate stage and develop to organoids [4]. (3) Air-liquid interface (ALI) method [9,10]. In this approach, the top layer of the cells is exposed to air, and the basal surface is in contact with the liquid medium i.e., the organoids are cultured in a gel matrix and their lumen is directly exposed to air instead of submerged in culture media. The ALI method is currently mainly used to produce kidney organoids [10] and intestinal organoids [9].

In the following sections of this review, we began by assessing the critical limitations of traditional *in vitro* models and current organoids models. In Section 3, we discussed advantages of microfluidics for cell culture, and highlighted the elements which we think would be particularly useful for organoid models. In Section 4, while discussing various organoids-on-chip studies, we highlighted which particular advantage/element of microfluidics has played a crucial role to make the model successful. In the last section of the manuscript, we summarized the future challenges in reproducibility, scaling-up, vascularization, maturation, pharmacokinetics/pharmacodynamics modeling, and getting clinical viable organoid models.

## 2. Limitations of Current Organoid Models

**Figure 1:**
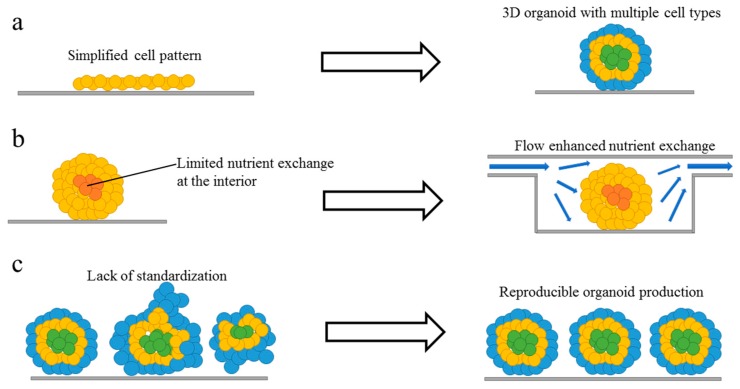
Limitations and goal of current organoid models. (**a**) Traditional *in vitro* models are too simplified, complex organoid models with multiple cell types and 3D architecture can be developed to better recapitulate *in vivo* organs. (**b**) There is a lack of nutrient exchange at the interior of the organoid, introducing flow and improving nutrient and gas exchange will help to create larger and more mature organoids. (**c**) Current organoid technology has limited uniformity and reproducibility, with better geometrical confinement and environmental control, future organoids production will be more reproducible.

In this section, we highlight some of the main limitations for traditional *in vitro* culture models and organoid models; these limitations need to be addressed when organoids are used for advanced developmental biology studies and drug screening applications (Figure 1).

**Problem 1**: Traditional *in vitro* culture models are too simplified to represent complex 3D tissues with multiple cell types (Figure 1a). This is due to the limited number of cell types and simplified environmental cues in the 2D models. Conventionally, cells are cultured with 2D tissue culture techniques *in vitro*. These simplified culture system has provided important and relevant insights into cell biology. However, in animal tissues and 3D cell culture models, cells and their surrounding microenvironment interact in all three dimensions [11] and enable us to have a better understanding of cellular behavior, both *in vitro* and *in vivo* [12]. When cultured as 3D models, cells exhibit features that are highly similar to the complex *in vivo* conditions and show significant improvement in terms of cell count, cell morphology, cell proliferation, and cell differentiation [13]. Due to the lack of 3D environmental cues, certain research questions, such as those relating to the structural development of neural tissues as well as neuro-degeneration, cannot be modeled and crucial questions not being properly answered using simplified 2D cell culture system [14].

Spheroids are one of the most commonly used *in vitro* 3D tissue culture models. They are formed by the aggregation of the cell and are often used in long-term culture [15]. Upon aggregating into spheroids, cells can establish contacts and create a microenvironment that allows the expression of tissue-like phenotypes [16]. However, most spheroid culture models contain only one cell type and do not completely capture the complex intercellular interaction between different cell types [17]. Compared with spheroids, organoids develop from stem cells or organ progenitors and self-organizes in a manner similar to *in vivo*. However, many current organoid models also do not take into account of blood cells or shear stress by blood flow, stroma, and immune cells [18]. Tumors found *in vivo*, for example, display complex genetic heterogeneity. On the other hand, it is still not clear whether the tumor organoids created *in vitro* recapitulates the original tumors and whether it harbors all the complexity as seen in the tumors *in vivo* [19]. Tumors *in vivo* often depend on the vascular network to deliver nutrients and oxygen, the angiogenesis process is stimulated by a variety of angiogenic mediators [20], this is not easy to recreate with existing *in vitro* models.

In existing 2D and 3D brain models, for example, there is often lack of microglia, the resident macrophages of the brain. Microglia are non-ectoderm (e.g., non-neuronal)-derived and are generally absent from the classic 2D cell culture systems or the more advanced 3D cell culture systems that go through a neuroectoderm intermediate. Importantly, microglia have been shown to be an important component in neurological diseases [21] and as a consequence brain organoid models without microglia lack a neuroinflammatory component that is essential for pathological events observed in human Alzheimer’s disease (AD) patients and AD mouse models [22]. Introducing microglia to the culture system will be beneficial if they are able to migrate in and take up residence as functional and ramified microglia.

**Problem 2**: Lack of nutrient/waste/gaseous exchange (Figure 1b)—One of the biggest obstacles in growing mature brain organoids is the restricted nutrient supply, gas exchange and waste removal at the interior of the organoids. The average diameter of organoids achieved in most studies is usually up to 3 mm [23,24] It is challenging to generate brain organoids in a biomimetic microenvironment favorable for brain development. Current brain organoid technologies are not capable of generating brain organoids that mature beyond the prenatal brain equivalent. In the process of brain organoid formation process, the EBs are initially encapsulated into Matrigel for culture [25]. Then, they are transferred into Petri dishes or spinning bioreactors for suspended culture to generate brain organoids. However, the major issue with the EBs is the lack of vascularization (even with the use of spinning bioreactor) which still limits the growth and maturation of organoids [26]. The brain organoid produced using this method mimics the initial human brain development but fails to show tissue maturation and complexity as seen in the adult human brain [4]. For many disorders and diseases that develop after the fetal development stage, the EB model is limited. Introducing perfusion flow through endothelial cells-lined channels to the organoid could prevent necrosis at the core of the organoids and enable the generation of a larger-sized organoid that develops past the prenatal stage type [27]. More complex organoids containing endothelial cells and blood vessel-like structures can also be created.

**Problem 3**: Lack of standardized organoids (Figure 1c)—Current organoid technology has limited uniformity and reproducibility, making it difficult to be used for toxicity screening or high-throughput testing [4,28]. This is due to inadequate engineering of the cellular microenvironment and the extra-cellular matrix (ECM). To use the organoids in drug screening or another medium to high-throughput applications, the organoids must be generated in a reproducible way [4]. Consistencies in the size and shape of the organoids will allow drug responses to be quantified more accurately. Organoids are conventionally generated in well plates and Petri dishes under static conditions [26]. Without any physical constraints or culture scaffolds, there is very limited control over the size and geometry of the organoid. The differences in the size, shape and cell numbers and their relative arrangement within each organoid leads to difficulties in normalizing the pharmacokinetics profile of drug candidates.

## 3. Developing Advanced Microfluidic Platforms to Improve Current Organoid Models

In 3D organoid models, as organoids increase in size and volume, the core becomes distant from the surface in contact with the fresh medium. Simple diffusion process provides insufficient oxygen and nutrient to the growing cells and limits the amount of waste being removed from cells in the core. Consequently, only cells in contact with fresh medium survive [29]. With microfluidic technology, tissue culture can be carried out in a controlled environment that optimizes temperature, pH, nutrient and oxygen supply and waste removal) [30,31]. Advances in microfluidic technology allow us to engineer the organoids with essential structural and physiological features in a controlled manner [11,32] and provide microscale structures and parameters that approximate the conditions *in vivo* [33]. Moreover, sensors and actuators can be integrated with the microfluidic devices to enable precise monitoring and control [34]. Optimization of the key parameters, such as the cell-cell and cell-ECM contact, cell type composition, tissue architecture, nutrient exchange, and various physical and electrical stimulation, may greatly minimize batch-to-batch variations and increasing fidelity.

When accompanied by microfluidic technology, 3D cell culture can be enhanced to become more complex organ-on-a-chip and organoid-on-a-chip models [31,35,36]. Microfluidic organs-on-a-chip platforms have been recently developed to create a variety of biomimetic organ models, such as lung [37,38], liver [31,39], kidney [40,41], heart [42,43] and neural networks [44]. They also allow integration of multiple tissue compartments to simulate multiple organs, and make systematic pharmacokinetics predictions for new drugs. Multi-organ chips have been made to capture the physiological complexity in the human body and study interaction between different organs [45,46,47].

It is also possible to use microfluidic approaches or substrates with different stiffness. These will subject cells to different forms of mechanical and physiological stress. Microfluidic platforms are often combined with flexible cell culture scaffold such as hydrogels. They are commonly used to create an *in-vivo*-like microenvironment for cell culture. They provide functional support for the cells–functioning as an extracellular matrix (ECM) while promoting survival, proliferation, and differentiation [48]. This is achieved by the natural property of hydrogels, which have interconnected pores with high water retention that allows nutrients to be transported to the cells efficiently. ECMs provide structural and biochemical support to the cells and promote cell-ECM interaction and growth [49,50]. Cell-ECM and cell-cell interaction can be tuned by changing the mechanical properties (for example stiffness) and compositions of each material in the scaffold [51]. Microfluidic approaches have advantages over other *in vitro* culture models since they offer better control over the physical and chemical parameters, the design of complex structures and the use of multiple materials to better mimic the *in vivo* organs. We summarize the advantages and disadvantages of each model in Table 1.

## 4. Organoid-on-a-Chip Models

**Figure 2:**
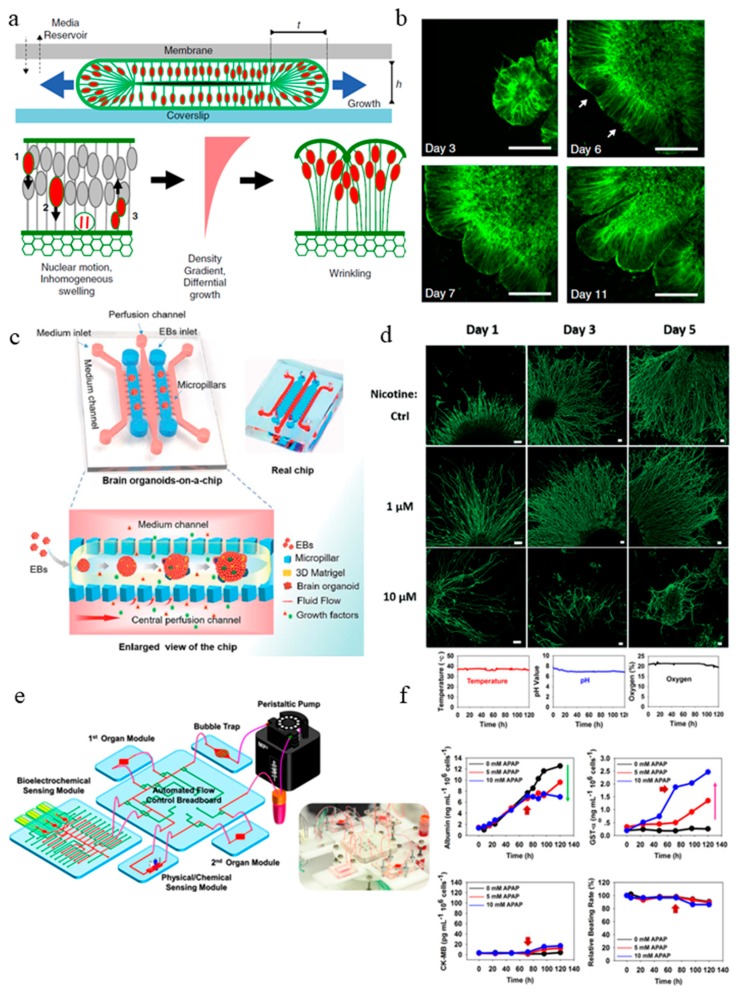

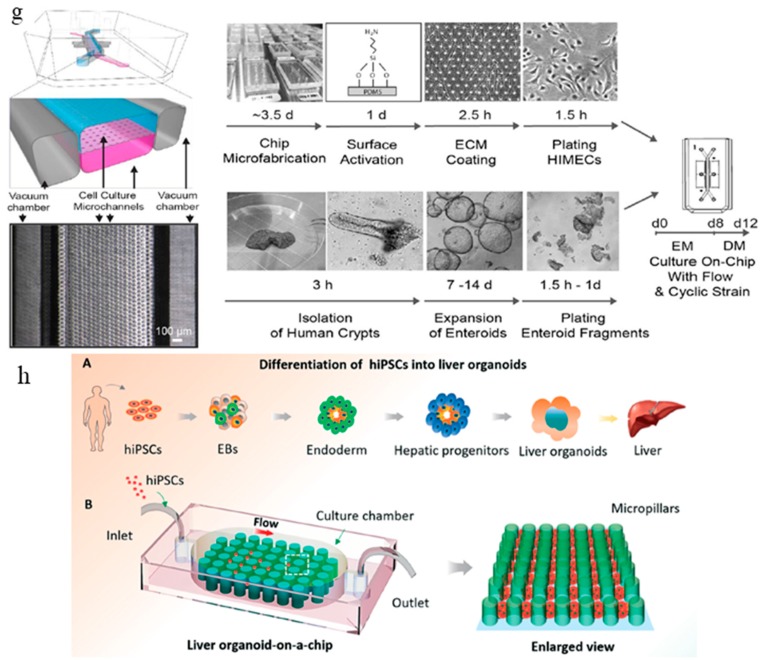
Current organoid-on-a-chip models. (**a**) Brain organoid development in the microchip compartment. The top membrane is coupled to a media reservoir, and the bottom coverslip enables in situ imaging. Wrinkling is caused by nuclear motion and position-dependent nuclear swelling. The differential growth leads to residual stress and wrinkling in the organoid [57]. (**b**) Fluorescence images showing the development of the organoid embedded in Matrigel, and the emergence of wrinkles. Arrows indicate initial wrinkling instability [57]. (**c**) The configuration of the brain organoids-on-a-chip device and the procedures for brain organoids generation on the chip. The EBs formed by hiPSCs were embedded in Matrigel, and the mixtures were infused into the culture channel. The EBs differentiated and self-organized into brain organoids [58]. (**d**) abnormal neurite outgrowth induced by nicotine exposure in the brain organoid-on-a-chip [60]. (**e**) The integrated microfluidic device consisting of modular components including microbioreactors, breadboard, reservoir, bubble trap, physical sensors, and electrochemical biosensors [61]. (**f**) Continual measurements of temperature, pH, and oxygen concentration within the integrated organoid-on-chips. The organoid-on-chip allows in-line automated electrochemical measurements of albumin and GST-α secreted from the hepatic organoids as well as CK-MB from the cardiac organoids. Beating analysis of the cardiac organoids can also be performed [61]. (**g**) Schematic representation of the intestine chip, showing the epithelial (blue) and microvascular (pink) microchannels separated by a porous PDMS membrane sandwiched in-between. The elastic membrane can be extended and retracted by the application of cyclic vacuum to the hollow side chambers. This actuation causes the mechanical deformation of the tissue layers cultured in the chip. (**b**) Procedure to establish the microfluidic co-cultures of the primary human intestinal epithelium and intestinal microvascular endothelium in the intestine chip [62]. (**h**) Generation of hiPSC-derived liver organoids *in vitro* and the configuration of the liver organoid-on-a-chip system. [58]. Reproduced with permission from [57,58,60,61,62].

Microfluidic organs-on-a-chip is an emerging 3D cell culture field that recreate the structural and functional features of human organs [36,52,53,54]. Organ-on-a-chip models have controlled fluid flow, cell-cell interaction, matrix property, as well as biochemical and biomechanical cues [37,55,56]. The organ-on-a-chip approaches enable development of organoids to millimeter diameter with enhanced nutrient exchange to prevent cell death at the core of the organoids. Karzbrun et al. for example studied the wrinkling and folding mechanism during brain development process with the help of microfabricated organoid-on-a-chip [57] (Figure 2a). They observed the appearance of convolutions after the organoid reached a certain cell density and nuclear strain. With the help of in situ imaging, they identified two opposing forces responsible for the differential growth in the organoid that leads to surface wrinkling: cytoskeleton contraction in the organoid core and nuclear expansion at the organoid perimeter. In the organoid-on-a-chip device, human embryonic stem cells (ESCs) were cultured inside the Matrigel-filled compartment, where they undergo self-organization into a shell-like structure with a lumen. In the course of weeks, the living organoids are sandwiched between a porous membrane and a glass coverslip, allowing nutrient to be exchanged and fluorescent live imaging to be carried out. They examined the nuclear motion and swelling during the cell cycle and quantitatively modeled the physics of the folding brain by using high-resolution imaging of single cells and image analysis (Figure 2b). In an experiment to treat the organoids acutely with blebbistatin, the actin cytoskeleton in the cells was disturbed. With this experiment, they concluded that cytoskeletal forces could maintain the organoid core contraction and provide the intracellular mechanical stiffness. They carried out a study to model a severe smooth brain malformation called lissencephaly *in vitro*. To induce the condition in the organoid, the LIS1 gene was edited with CRISPER/Cas9 genome editing. Modified ECM and cytoskeleton formation and reduced cell elasticity were observed in the LIS1 mutant organoids. In this study, the use of a microfabricated-chip allowed the organoid to be supported by a Matrigel scaffold while being physically confined in a small compartment to allow wrinkling and folding to initiate. At the same time, diffusion of nutrient and waste could occur through the porous membrane. This is otherwise unachievable in the conventional organoid model. Overall, the organoid-on-a-chip was demonstrated to be a useful model to study the biological and biophysical mechanism of brain development.

Another microfluidic brain organoid-on-a-chip model was developed recently by Wang et al., the organoid culture and differentiation were done in situ under perfusion culture [58] (Figure 2c). Compared with other organoid generation methods, where cells from EB differentiate into organoid in 3D suspended culture [4,59], this organoid-on-a-chip creates a platform that allows the EB to mature into self-organized organoids under a controlled microenvironment. The system recapitulates the *in vivo* organ by incorporating 3D culture Matrigel scaffold, dynamic perfusion flow, and multicellular heterogeneous tissue architecture. Similar to the conventional organoid generation method, organoids cultured on this microfluidic platform exhibited well-defined neural differentiation, regionalization, and cortical organization. Compared with 2D-cultured organoids, the perfusion-cultured organoids expressed enhanced levels of cortical markers (TBR1 and CTIP2), indicating differentiation and progression of organogenesis.

In a follow-up study by the same group [60] (Figure 2d), the same microfluidic brain organoid platform mentioned above was used to examine the effect of prenatal nicotine exposure on brain development. The organoids exhibited typical features of a fetal brain during development. When subjected to nicotine exposure, premature neuron differentiation and abnormal brain regional growth were observed. The chip allowed in situ tracking and imaging of the organoid, enabling the visualization of the brain regionalization process as well as the monitoring on the chip the nicotine exposure-induced premature neural differentiation by immunohistochemical staining.

The field of organ-on-a-chip has been around for almost 10 years [37]. Although a variety of microfluidic organ-on-a-chip devices with biomimetic compositions, architecture, and functions have been created, there are limited efforts in using reporters and microsensors to monitor different cellular parameters such as growth or metabolism and changes in the culture in the system. This is especially important for drug testing studies in organ-on-a-chip devices which requires an extended period of culture time. To bridge this gap, Zhang et al. developed an integrated modular platform with fluidics-routing breadboard as well as physical, biochemical and optical sensing capabilities [61] (Figure 2e). They demonstrated the generation of liver and heart organoids on this platform and validated the capability of the in situ biosensors. The whole platform was encased inside a customized benchtop incubator, including the automatic pneumatic valves for fluidic control, the electronics for physical sensors, and a potentiostat controlled by a centrally programmed computer. The microfluidic device consists of a number of modules: microbioreactors, breadboards, reservoir, a bubble trap, physical sensors, and electrochemical sensors. The label-free immunobiochemical sensors monitor the soluble biomarkers secreted by organoids (Figure 2f); the detection is based on the change of interfacial electron transfer kinetics of redox probe [Fe(CN)_6_]^4−/3−^ upon antibody-antigen binding. Upon saturation of captured antigen binding, they adapted a mechanism to regenerate the electrode surface so it can be reused for subsequent measurements. The immunobiosensing mechanism developed here is a promising tool because by using different antibodies, the functionalized biosensors were able to measure a wide range of soluble biomarkers secreted by the organoids. Apart from the biochemical sensor, a physical sensing unit to monitor temperature, pH, and oxygen levels were installed as well (Figure 2f). Inside the microbioreactor, the organoids were constructed using a micropatterning technique through a photo mask. Cells were then encapsulated in gelatin methacryloyl (GelMA) and loaded in the microbioreactor to form spheroid *in situ*. Finally, they demonstrated that the system could perform automated sensing of drug-induced organoid toxicity using the biochemical sensors at various drug concentrations. This work showcases efforts towards the adaptation of organoids-on-a-chip for in situ organoid generation and automated drug testing.

The intestine organoid culture models derived from Lgr5^+^ intestinal stem cells are usually embedded within Matrigel [3]. They can be propagated indefinitely under growth factor treatment. The organoid model recapitulates the heterogeneity of the intestinal epithelial layer and is suitable for in situ imaging of intestinal development. However, the presence of an enclosed organoid lumen is non-physiological, since secreted biochemical and waste products concentrate within the central lumen instead of being transported through peristalsis and luminal flow. Moreover, as mentioned in the previous section, organoid cultures lack tissue-tissue interfaces, mechanical stimuli, and interaction with other cell types such as immune cells. To this end, Kasendra et al. developed a more complex and physiologically relevant microfluidic intestinal culture system using human cells derived from organoids generated from biopsies of the human intestine [62]. This microfluidic chip enables 3D intestinal villi-like structures to be formed in situ. This chip contains two culture channels: an epithelial channel and a vascular channel. They are separated by a thin (50 μm) flexible ECM-coated porous PDMS membrane containing 7 μm diameter pores (Figure 2g). The epithelial and vascular channels are flanked on either side by two hollow vacuum chambers that permit application of cyclic suction to mechanically stretch and relax the microchannels to emulate the peristaltic motions of a living human small intestine. The epithelial cells cultured on the chip exhibited multi-lineage differentiation, established epithelial barrier function, and produced mucus. The transcriptome analysis of the cells cultured on the intestine chip more closely resembled that of adult human duodenum *in vivo* than the original organoids that were used to generate the cells to seed in the chips. The study exemplifies an improvement from past *in vitro* intestine microfluidic models [63,64] by incorporating primary cells isolated from patient-specific biopsy-derived organoids as well as gut microvascular endothelial cells. The model supports the formation of villus-like structures without the need to use a micropatterned villus-shaped flexible scaffold.

Microfluidic chips can also be used as a simple and robust strategy to generate organoids. Wang et al. fabricated a micropillar chip perfusion system to generate liver organoids from EBs. The hiPSCs went through hepatic differentiation and maturation in situ (Figure 2h) [58]. A sizable number of human hepatic organoids can be generated this way in a straightforward and controlled way. To control the size and uniformity of the EBs and organoids, the dimensions of the micropillar array were optimized. This micropillar chip array method allowed the formation of uniform EBs and generation of liver organoids afterward in a confined space, avoiding the tedious procedures in the conventional organoid generation methods. The media perfusion in the culture chamber facilitated nutrient transport and waste removal. This study is an excellent example of how engineering approaches using microfluidic chips can address the issue of lack of standardization in conventional organoid generation processes.

## 5. Conclusions and Future Perspective

Organoids have helped to expand our understandings of human organ development and have become a useful tool to model human diseases and test therapeutics *in vitro*. In addition, they contribute to reducing the use of animal models and cost in the pharmaceutical industry. Despite recent developments in the field of organoid-on-a-chip, the full potential of the microfluidic organoid platforms is yet to be realized but will further improve with more refined technical innovations. Limitations of current organoids, which are still highly simplified models of human tissues or organs, may be improved by incorporating microfluidic platforms. The lack of nutrient and gas exchange hinders the maximum size and extent of tissue maturation of the organoids. Exposure of cells to physiological shear flow, mechanical stress, and substrate stiffness can have profound effects on cell and tissue physiology. There is still limited control over the size, shape and relative arrangement of different cell types within the organoids in 3D format, limiting their applications for reproducible quantitative studies as required for robust drug screening and testing.

We envision that the next generation of organoid-on-a-chip will have the potential to be scaled-up to be suitable for high-throughput analysis and commercial applications. This will require the generation of reproducible organoids with respect to cell-type composition and arrangement, and overall organoid structure and organization. To increase the critical size of the organoids, more efforts will be made towards promoting vascularization of organoids, in the form of artificial blood vessels in the organoids or spontaneously generated vessels due to activation of angiogenic pathways in incorporated vascular cell types. In order to incorporate multiple organoid types into single chips, the culture medium and physical conditions in the microfluidic platform may need to be optimized for each organoid type. The ultimate goal for microfluidic multi-organoid systems is to achieve *in vivo*-like settings that capture the structure and physiology of the different organ systems and recapitulate inter-organ interactions and crosstalk.

Future engineering approaches will enable organoid models to be utilized in clinical trials and elucidate personalized pathology mechanism and drug responses. To realize this, the time needed to generate fully functional organoids need to be shortened. Currently, it can take months to build an organoid from stem cells derived from primary tissue, this is too long for clinicians to make effective decisions. Due to the difference in systemic drug distribution profile and immune responses that are not observed *in vitro*, *in vivo* drug responses are often different from those determined *in vitro*. More extensive engineering and screening effort are needed to provide proper information about physiologically relevant pharmacokinetic/pharmacodynamics (PK/PD) profile. We believe that appropriate clinical tissue model can be created by combining the ability of microfluidic platforms to precisely control drug input and distribution, with that of organoid models to recapitulate tissue organization and function.

## Figures and Tables

**Table 1:** Comparison of advantages and disadvantages of microfluidic chips and other *in vitro* culture models.

*In Vitro* Culture Models	Advantages	Disadvantages
2D cell culture (culture dish, transwell membrane, and culture flask)	Well established protocol Easy to handle and quantify	Static condition Lack of physical and biochemical chemical cues Large media volume Large variation in nutrient and waste concentration
3D cell culture (engineered culture scaffold, spheroid, microcarrier, tissue biopsy, organoid)	Include cell-cell and cell-ECM interaction Capture the 3D architecture of tissue culture Sensitive to drug treatment	Static condition Inefficient nutrient and waste transport
Microfluidic chip (Organ-on-a-chip)	Fine control over microenvironment Good mass transport provided by fluid flow Ability to integrate with various sensors and actuators	Difficult to standardize and scale up Require external pumps, tubing, connectors, and valve to operate
